# Collective Dynamics of Gene Expression in Cell Populations

**DOI:** 10.1371/journal.pone.0020530

**Published:** 2011-06-15

**Authors:** Elad Stolovicki, Erez Braun

**Affiliations:** Department of Physics and Network Biology Research Laboratories, Technion-Israel Institute of Technology, Haifa, Israel; Ludwig-Maximilians-Universität München, Germany

## Abstract

The phenotypic state of the cell is commonly thought to be determined by the set of expressed genes. However, given the apparent complexity of genetic networks, it remains open what processes stabilize a particular phenotypic state. Moreover, it is not clear how unique is the mapping between the vector of expressed genes and the cell's phenotypic state. To gain insight on these issues, we study here the expression dynamics of metabolically essential genes in twin cell populations. We show that two yeast cell populations derived from a single steady-state mother population and exhibiting a similar growth phenotype in response to an environmental challenge, displayed diverse expression patterns of essential genes. The observed diversity in the mean expression between populations could not result from stochastic cell-to-cell variability, which would be averaged out in our large cell populations. Remarkably, within a population, sets of expressed genes exhibited coherent dynamics over many generations. Thus, the emerging gene expression patterns resulted from collective population dynamics. It suggests that in a wide range of biological contexts, gene expression reflects a self-organization process coupled to population-environment dynamics.

## Introduction

Understanding the emergence and maintenance of stable cellular phenotypes and the switching of phenotypes in response to environmental changes is at the forefront of biological research in diverse areas of study such as cancer and development. It is well known that identical genotypes can develop into diverse phenotypes. Moreover, isogenic cells in the same environment may exhibit some degree of phenotypic variability [1], [2], [3], [4], [5], [6], [7] and can even switch between two well-determined phenotypes [8], [9], [10]. However, given the apparent complexity of genetic networks, an open question is: what are the processes leading to the stabilization of a particular phenotypic state of a cell—its morphology, metabolism and function? Many years ago, Waddington coined the concept of “epigenetic landscape”, in an attempt to construct a useful metaphor for the underlying complex mechanism of genotype-to-phenotype transformation [11]. Waddington proposed that “strategic” principles, beyond individual molecular interactions, were required for bridging the gap between the short-term physiological responses and the long-term evolutionary processes and this, he believed, will come through understanding the epigenetic landscapes underlying developmental processes (e.g., cell differentiation). Notwithstanding the impressive progress in molecular biology over the last decades, the strategic relation between the process of gene expression and the emergence of specific phenotypes has remained elusive.

On the one hand it is commonly thought that the emergence of a stable phenotype is the result of a programmed system, in which, for a given genome and environment, a set of “instructions” for the phenotype is instilled (via evolution) in the underlying genetic regulatory circuits [12], [13], [14]; phenotypic variability is then the result of unavoidable “noise”. On the other hand, given the huge combinatorial phase-space spanned by the gene-expression degrees of freedom [15] and the significant levels of intracellular and environmental fluctuations [10], it has been proposed that stable phenotypic states emerge as attractors in the phase-space determined by the concentrations of expressed proteins; given a genetic network architecture (connectivity), the finite number of attractors guarantees the stabilization of specific phenotypes by dynamically directing the initial vector of expressed proteins into one of its stable steady states [15]. This attractive concept, a modern version of Waddington landscape metaphor, was developed theoretically within the framework of specific models and for certain classes of networks it was shown that attractors do emerge naturally in the system, i.e. they are properties of the network's connectivity and structure [15], [16]. However, many questions related to the attractor idea remain open: what intracellular processes do actually determine the stable attractors and their basins of attraction [17], [18]? Do these attractors reflect the intrinsic dynamic response of genetic networks to environmental signals? What is the level of degeneracy in the phase-space of expressed genes? Do many different attractors result in similar cell phenotypes? The experimental basis necessary to tackle these key issues is still lacking [19]. In this paper we attempt to advance our understanding on these matters by studying the relation between the emergence of a stable phenotype in response to an environmental switch and the underlying gene expression dynamics.

Biological cells are history-determined systems, so understanding their intrinsic dynamics requires us to discriminate between necessity and contingence, between inevitable and accidently-instilled intracellular processes in evolution. Toward this end, we have studied the gene expression response of cell populations adapting to surmount a severe unforeseen challenge. Since it is unlikely that genomic circuits are “pre-programmed” to respond to any arbitrary novel perturbation, an unforeseen challenge has the potential of exposing gene expression patterns beyond the specific ones shaped by evolution [20], [21], [22]. Here, we utilize this approach as an effective tool to probe the expression dynamics underlying the emergence of a novel stable phenotypic state of the cell. To gain further insight on the emerging dynamic patterns of expression we study for comparison, “wild-type” cell populations responding to a common environmental switch. The fascinating process of adaptation to a severe unforeseen challenge was discussed in our previous publications [20], [21], [23] and is not the focus of the current paper. This setting of populations adapting to a challenge however, provides us with an effective model system to study long-term population expression dynamics throughout phenotypic changes that were unlikely to be “pre-programmed” into the cellular regulatory system.

Experiments were performed on unicellular yeast cells in which the essential gene *HIS3* from the histidine biosynthesis pathway was detached from its natural regulatory system and was placed under the exclusive regulation of the GAL system responsible for galactose utilization [21]. “Wild-type” cells with identical genomic background but deleted of the gene *HIS3* were used for comparison. The arbitrary *HIS3* rewiring, linking the foreign histidine and GAL systems, was shown to be stressful and challenging by creating incompatibilities in gene expression [20]. In particular, a switch from a galactose-based to a glucose-based, histidine-lacking medium presented a severe unforeseen challenge to the cells since the GAL system and the GAL-controlled *HIS3* were initially strongly repressed in glucose. Note that cells deleted of *HIS3* could not survive in a medium lacking histidine [21]. Recently, we have shown that a cell population carrying this GAL-*HIS3* rewired genome could rapidly adapt (within ∼10 generations) to grow competitively in this medium despite the strong initial repression of *HIS3* [21]. Similar adaptation of genome-rewired cells to glucose was shown for different culture techniques: chemostats, batch cultures as well as for cells grown on agar plates [21], [23]. Once established, the adapted state had been propagated stably for hundreds of generations. Our previous work showed that the inherited adaptation *was not due to selection*; every cell in the population had, in principle, the potential ability to adapt [23]. Indeed, we have shown that the adaptation was due to a response of many individual cells to the glucose medium and not due to selection of rare advantageous phenotypes.

Intriguingly, underlying the adaptation process was a global re-organization of gene regulation. We have previously shown that the adapting cell populations exhibited genome-wide expression dynamics involving a sizable fraction of the genome and presented strong correlations between genes across functional modules [20]. These results revealed that co-expression does not necessarily imply co-functionality. Moreover, the observed crosstalk between functional modules presumably played an important role in enabling the emergence of a proper metabolic state. We also observed the simultaneous induction and repression response of genes residing within the same functional metabolic module. Thus, co-functionality does not necessarily imply co-expression and there is no simple connection between transcriptional patterns and metabolism. Importantly, the global gene expression response was found to be non-reproducible between repeated experiments that nevertheless showed similar population growth dynamics and metabolism [20]. This is a surprising result, since the irreproducibility in expression patterns was global and spanned the entire set of metabolic genes participating in the emergence and maintenance of a stable adapted growth phenotype. These results indicate that a spectrum of different gene expression patterns can potentially arise in populations under the same experimental conditions.

Gene expression response and its relation to the phenotypic cell state depend both on the environment and the history of the population. Thus, it is difficult to exclude the possibility that the variability in gene expression, even between isogenic populations grown in the same environment, results from their different histories. In this paper we overcome this problem by an experimental approach that enabled us to probe the gene expression patterns underlying phenotypic order through studies of the population dynamics while controlling for environmental conditions and population history. To compare the dynamics of gene expression between populations with identical histories, we developed a novel experimental setup in which two populations with a joint history could be separated at a defined time point and examined under identical environmental conditions. Our genome-rewired cell populations were grown in chemostats under severely challenging conditions in which cells fiercely competed for limited resources. Thus, the relevant phenotype that integrates essential metabolic functions was that of growth rate and proliferation and this phenotype was highly constrained for the adapting cells in our experiments.

The results of the present paper advance our previous work in two important aspects. First, we show here that chemostat populations with *identical histories* nevertheless demonstrated variable expression dynamics of essential genes. Second, by utilizing high temporal resolution, low-noise gene expression measurements, the set of experiments presented here show that the observed variable gene expression patterns were not due to cellular “noise”. Rather, these patterns of expression reflected *collective dynamics* resulting from synchronization of the expression response of the cells within the population. Thus, the population itself was the proper level of organization determining the cellular gene expression response via its collective dynamics. We demonstrated the generality of this mechanism by showing collective dynamics of gene expression also for “wild-type” cells.

## Results

### Response dynamics of rewired cell populations

To construct two populations with the same history, two identical chemostats, initiated from a single clone of GAL-*HIS3* rewired cells, were coupled via an external pump so that their cell content was mixed at a rate much faster than their dilution rate (see *Methods*). A steady state was first stabilized in galactose for these coupled chemostats, after which the mixing of cells between them was stopped, they were decoupled so each one contained its own isolated population, and their common feeding medium was switched to glucose. Thus, after decoupling, the initial single galactose steady-state population was separated into two “twin” populations, allowing comparison of their separate responses to the medium switch into glucose. Note that both chemostats were fed from the same source of medium which provided identical feedings for the twin populations. Since the mixing of cells between the coupled chemostats prior to the switch to glucose was much faster than their dilution rates, as long as they were coupled they effectively contained a single population as the fast mixing caused the same cells to pass several times back and forth between the reactors before being diluted out. The cell density in the chemostat, in particular during the epoch of cell adaptation, is a sensitive function of the integrated metabolic reactions contributing to growth and proliferation [21], [24] and thus, served as a measure for the average phenotype of the cells.

**Figure 1:**
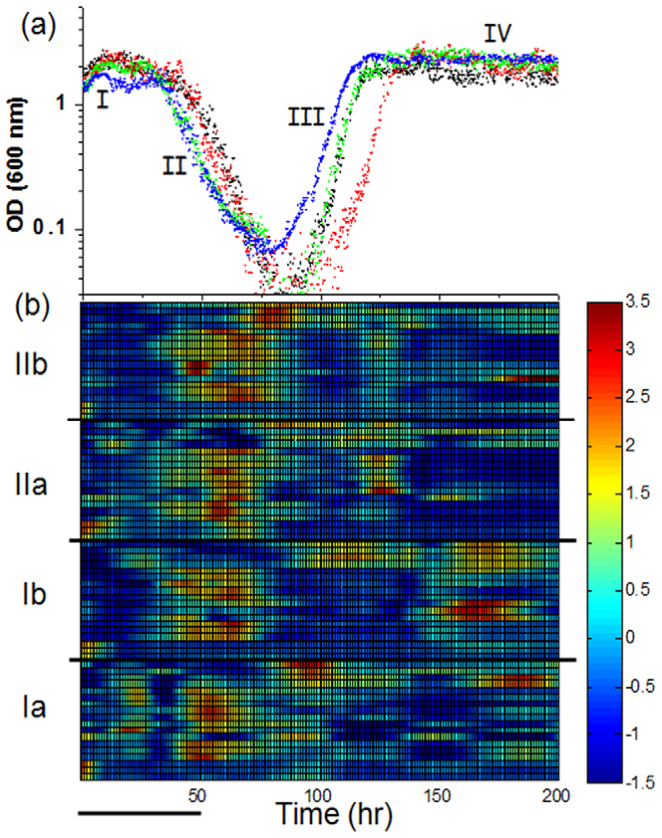
Phenotypes and gene expression profiles. (a) Cell density (OD at 600 nm) as a function of time for two pairs of twin chemostats with populations of rewired cells (Ia-black and Ib-red are twin populations and so are IIa-blue and IIb-green). The histidine-lacking medium was switched from galactose to glucose as a sole carbon source at t = 0, leaving all other nutrients the same. A steady state typical of galactose metabolism was first established as a single population for each pair of twin chemostats which were decoupled prior to this medium switch into glucose. Note the y-axis logarithmic scale. Different phases of the dynamics are marked I–IV. (b) Color-coded raster plot of the mRNA expression profiles: Ia–Ib and IIa–IIb mark the same twin populations as in (a). The expression levels were measured for 18 genes belonging to different metabolic functional modules (see *Methods* for list of genes at the same order of appearance as in the figure for each population, starting with *HIS3* as the first gene from the bottom). The measured expression levels were normalized for each gene to zero mean and unit standard deviation across its entire time profile. The color-coded profiles are cubic-spline interpolations of the measured data points shown in Fig. 2. Bar - 10 chemostat-dilution generations.

Fig. 1a shows the cell density as a function of time following the switch from galactose to glucose (t = 0; after decoupling of the twin chemostats), for two pairs of twin populations. The growth dynamics were similar in all cases and were composed of four distinct phases [21]: (I) an exponential increase in cell density, followed by (II) a sharp exponential decline in density which then, (III) turned again into an exponential increase and finally, (IV) stabilized at a new steady state. Twin chemostats exhibited higher similarity in phase II than populations from separate experiments. Phase II is crucial, since as we have shown before, cells became fully adapted to grow on glucose during this phase [23]. The most significant variation between populations developed in phase III—the recovery of the already adapted cells to the chemostat steady state condition, but eventually all populations stabilized at approximately the same steady-state cell density. Thus, the population dynamics were weakly history-dependent but the dispersion between the population-average metabolic states was minor. Note in Fig. 1a that the metabolic response of the twin populations during phase II was identical, which was not the case between populations with different histories. This proves that any putative small differences between the reactors, if existent had a negligible effect on the metabolism and the population dynamics. As was shown in detail in [21] there was a significant population growth and cell division during phase II, allowing eventually population adaptation to glucose. The decline in cell density along this phase, reflects an average cell growth-rate lower than the chemostat dilution rate and not merely dying cells. This was manifested in an exponential decline slower than the chemostat dilution rate and was also verified by direct microscopy imaging of cells along this phase [21].

Given their identical history and similar metabolism, how similar are the populations gene expression dynamics? We measured the transcriptional expression dynamics in conjunction with the growth dynamics at high temporal resolution in parallel populations sharing an identical history. Expression at the level of mRNA molecules (transcription) served as a proxy to the regulatory dynamics. Fig. 1b depicts in a color-coded raster plot the normalized mRNA profiles of these populations, which include 18 genes belonging to four different metabolic groups: GAL genes (plus *HIS3*), Histidine, Purine and Glycolysis pathways. These genes were chosen since under our experimental conditions, together with *HIS3*, they were absolutely essential. Outside of the GAL system which responds by strong repression to the switch into glucose, these metabolic groups are not weak factors in glucose metabolism [25], [26], [27]. Correspondingly, these genes were found to respond strongly in our previous genome-wide measurements of adapting rewired cell popolutions [20]. Cell growth is thought to be a sensitive function of the expression of metabolic genes participating in the relevant biochemical modules [28]. Thus, under our experimental conditions and in particular during adaptation to a severe challenge, one expects that the level of expression of these metabolic genes would be constrained by the cellular metabolic requirements. Common to all populations was the emergence of activity peaks within phase II, long after the transition to glucose (∼50 hrs ∼10 chemostat-dilution generations). Additional activity peaks appeared at later times. These peaks of activity were significant: these responses were much larger than the measurement errors (see below detailed analysis of the responses).

Two features in the data deserve particular attention: first, consistent with our previous genome-wide measurements [20], each of the populations developed its own unique expression pattern. In particular, both pairs of twin populations that shared identical history and experienced identical external conditions displayed significantly different patterns of expression. This intriguing result shows that identical histories did not guarantee similar population-average expression patterns, despite the similarity in population growth dynamics. Second, the relaxation time of an expression peak was much longer than the cell generation time. As such, the activity peaks emerging in populations of 10^8^–10^9^ cells, ∼10 chemostat-dilution generations after the medium switch perturbation must be an outcome of *collective dynamics* requiring some sort of coupling between the cells; stochastic cell-to-cell fluctuations would be averaged-out in such large populations. Note that the long time-gap between the medium switch into glucose and the emergence of the expressions activity peaks (∼50 hrs), means that the latter was not a direct response to the environmental perturbation but rather reflected population dynamics. Moreover, the broad expression peaks spanned more than an order of magnitude in cell density without losing phase coherency. Since cells exhibited significant growth during phase II [21], these expression dynamics required a high degree of coherency during cell division, preserving the correlated dynamics along generations.

**Figure 2:**
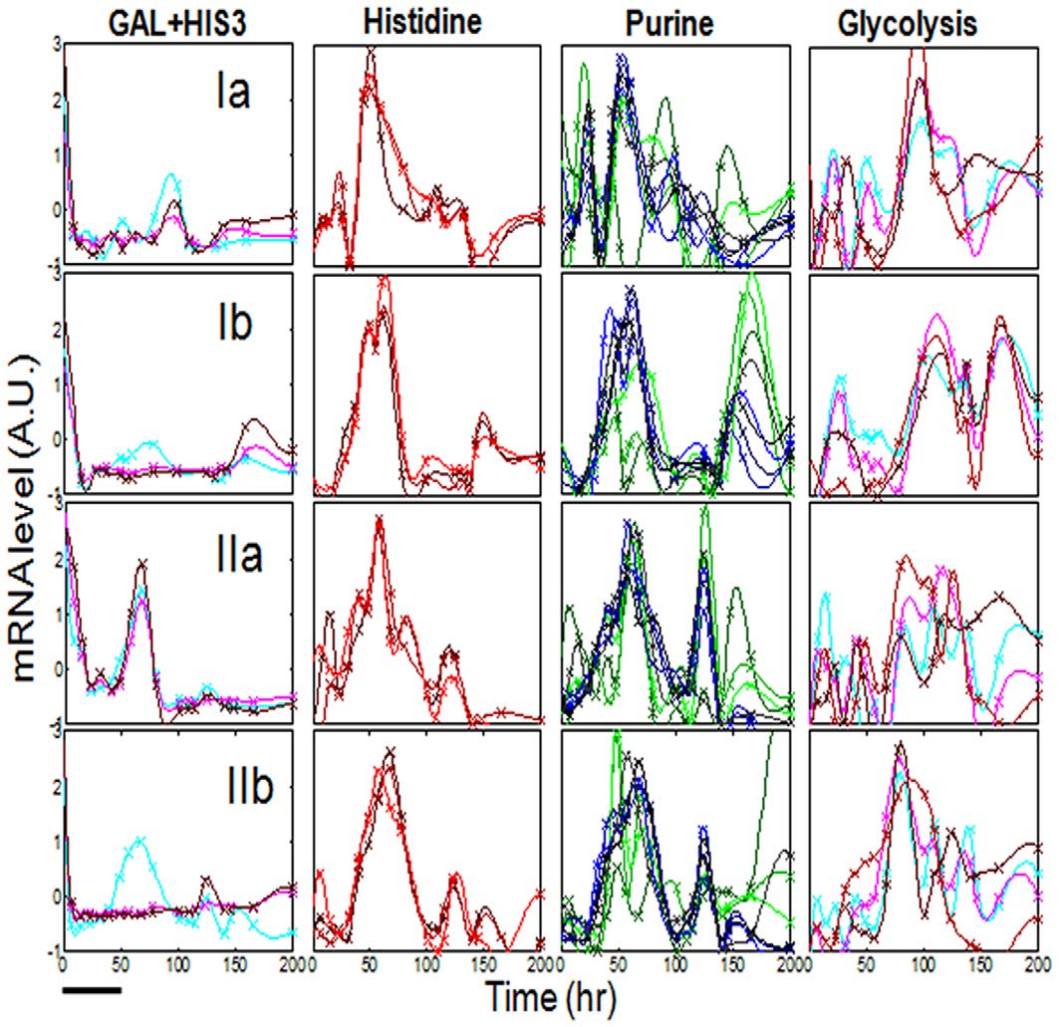
Expression profiles. The normalized mRNA expression levels for the four populations of Fig. 1 (each row is a different population as marked). The same genes shown in Fig. 1b were separated by their functional annotation groups (different columns): GAL genes plus *HIS3* (Left column, the rewired *HIS3* gene is in cyan), Histidine pathway (second column from left), Purine pathway (third column from left) and Glycolysis (right column) (see *Methods* for the list of genes). The measured mRNA profile for each gene (relative to the value of *ACT1* at that time point) were normalized as in Fig. 1b, by subtracting the mean value and dividing by the standard deviation; mean and standard deviation were computed over the entire measurement period. The lines are cubic-spline interpolations of the data points. The medium was switched from galactose to glucose at t = 0. Bar - 10 chemostat-dilution generations.

Fig. 2 compares the normalized expression profiles of the genes according to their different functional groups between the four populations (un-normalized profiles are shown in Fig. S1). Note that genes belonging to the same functional group may exhibit different dynamics [20]. In particular, the rewired *HIS3* gene might or might not exhibit similar dynamics to the GAL genes (Fig. 2, left column). Higher resolution measurements (Fig. S2), revealed possible higher frequency modes but basically retained the main features observed in Fig. 2. Neighboring time points measured from cells extracted separately from the chemostat, show that measurement errors were insignificant compared to the measured activity peaks; error analysis using bootstrap resampling is presented below. Repeated measurements assessing the errors arising from the real-time PCR technique itself are presented in Fig. S3, showing that technical replicates exhibited negligible errors.

### Correlations within and between populations

**Figure 3:**
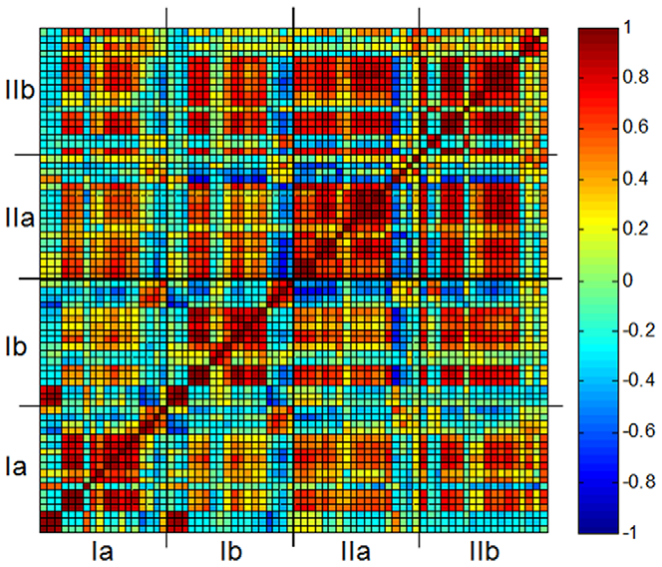
Correlation coefficient matrix. The Pearson correlation coefficient between the mRNA time profiles shown in Fig. 1b, computed for all pair of genes in the four populations (see *Methods* for the definition and computation of the correlation coefficient). For each gene-pair, the correlation coefficient is the result of averaging over the entire period shown in Fig. 1. The correlation patterns are insensitive to the averaging time interval. Randomly-shuffled surrogate profiles showed zero correlation coefficients. The order of genes for each population is the same as in Fig. 1b. Near-diagonal pixels depict correlation coefficients within populations, while off-diagonal pixels are between populations (populations marked as in Fig. 1).

To quantify the inter-gene correlations in expression dynamics, Fig. 3 shows the pair-wise correlation coefficients between all measured genes. The Pearson correlation coefficient for each pair of genes, within and between populations was computed as the zero-lag normalized covariance values of the measured expression profiles over the entire set of measured time points shown in Fig. 2 (see *Methods*). We found significant inter-gene correlations within each population. Inter-population correlations were also significant (off-diagonal elements), but weaker than the intra-population ones (near-diagonal elements). Clearly, correlations were not necessarily higher between twin populations compared to populations with different histories. The stability of the gene expression patterns over time is apparent from the significant intra-population correlations. This stability also testifies to the stability of our measurement setup. This is consistent with our previous results discussed in [20] showing that the global patterns of expression were stable in our chemostat setups over hundreds of hours (>50 generations).

**Figure 4:**
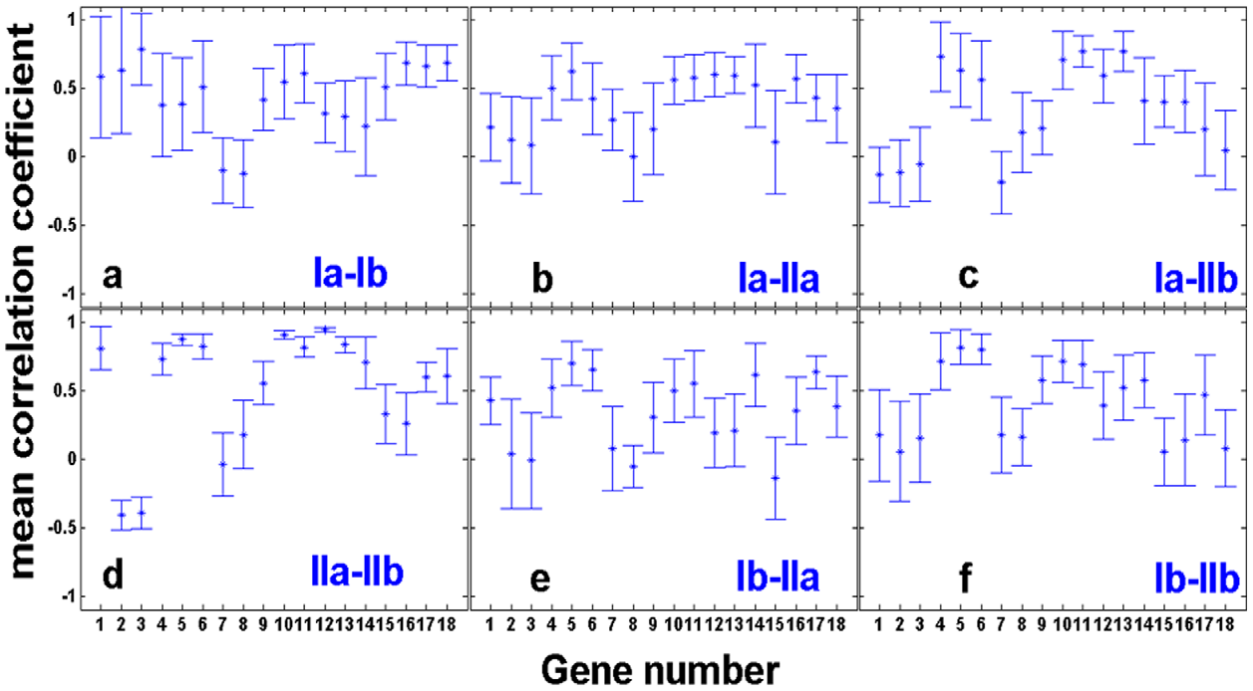
Bootstrap correlation coefficients across populations. Mean and standard deviations of correlation coefficients computed between a given gene in one population and the same gene in another population. Bootstrap resampling (see *Methods*) was used to compute the mean and standard deviation (error bars) of the correlation coefficients for genes between populations: (a) Ia and Ib, (b) Ia and IIa, (c) Ia and IIb, (d) IIa and IIb, (e) Ib and IIa and (f) Ib and IIb. Note that (a) and (d) show the correlations between twin populations. The gene number on the x-axis is at the same order as in Fig. 1b and corresponding to the list presented in *Methods*.

We used bootstrap resampling to further characterize the correlations and their statistical significance for each individual gene pair, within and between populations. Figs. 4a–f show the correlation coefficients between a gene in a given population and the same gene in all other three populations, computed from the bootstrap resampling data (see *Methods*). The bootstrap analysis verified that there were significant correlations between populations for some genes but not for others and even anti-correlations between populations for some of the genes. Moreover, it shows that beyond errors, twin populations (Fig. 4 left column; a and d) did not necessarily exhibit higher correlations than populations with no common history. As a control, Fig. S4a shows similar patterns in the correlation coefficients matrix for one pair of twin chemostats measured at higher temporal resolution (expression profiles shown in Fig. S2).

Measurements on mRNA samples extracted from cells collected from the chemostat at neighboring time points to the ones presented in Fig. 2, is a way to estimate the errors between “biological replicates”. Given the inherent irreproducibility of expression patterns between repeated chemostats, this was the only practical way of estimating the errors arising in biological replicates. Fig. S4b compares the correlation coefficients between a gene in population Ia and the same gene in population Ib using bootstrap resampling data based on the expression profiles of Fig. 2 (the same as in Fig. 4a; red) and the ones based on the higher resolution profiles which include the extra neighboring time points of Fig. S2 (black). The two sets of data exhibited similar results and similar errors and thus sampling the population expression dynamics at more time points would not affect our conclusions.

**Figure 5:**
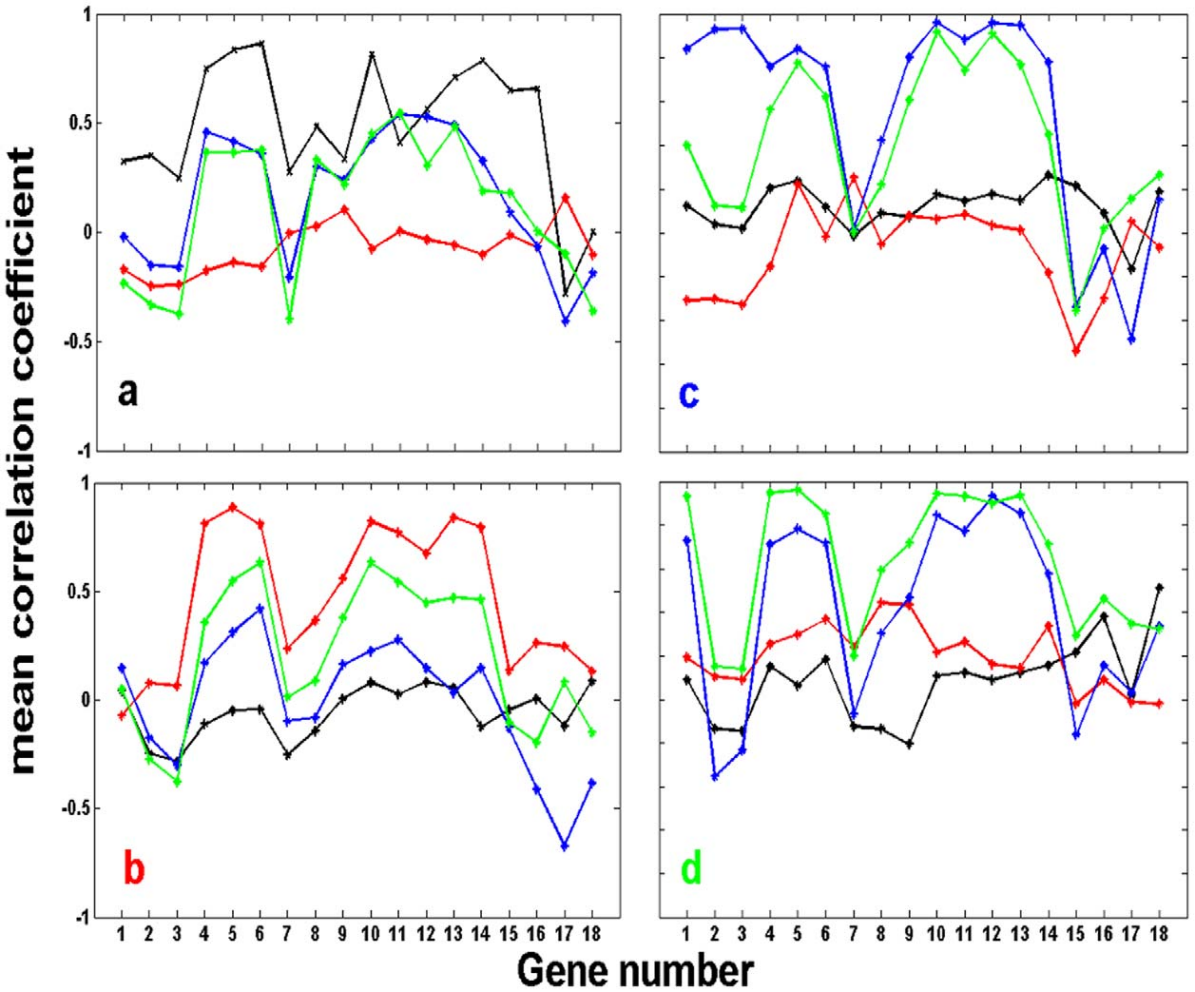
Mean correlation coefficients between all genes within and between populations. The bootstrap resampled data was used to compute the mean correlation coefficients between a gene (corresponding to the number on the x-axis; numbering at the same order as in Fig. 1b and according to the list presented in *Methods*) from a given population, and all other genes within the same population and between populations, taking errors into account (see *Methods*). Each figure shows the correlation coefficients between a gene from population (a) Ia, (b) Ib, (c) IIa, and (d) IIb and all the genes within and between populations according to the following colors: population Ia (black), population Ib (red), population IIa (blue), and population IIb (green).

Bootstrap resampling also allowed us to assess the mean correlation coefficients across all genes within and between populations, taking into account errors (see *Methods*). Figs. 5a–d show the mean correlation coefficients between a gene (whose number is depicted on the x-axis ) from a given population: (a) Ia (black), (b) Ib (red), (c) IIa (blue), and (d) IIb (green), and all genes from the other populations, according to the specified color scheme. Note that a curve with the same color as the plot-label represents the mean correlation coefficient between a given gene and all other genes within the same population. Fig. 5 clearly shows the emergence of significant correlations for some of the genes and lack of correlations for others. It also verifies the result apparent in Fig. 3; significant correlations between some of the genes in different populations but not necessarily stronger correlations between the twin populations sharing a common history.

**Figure 6:**
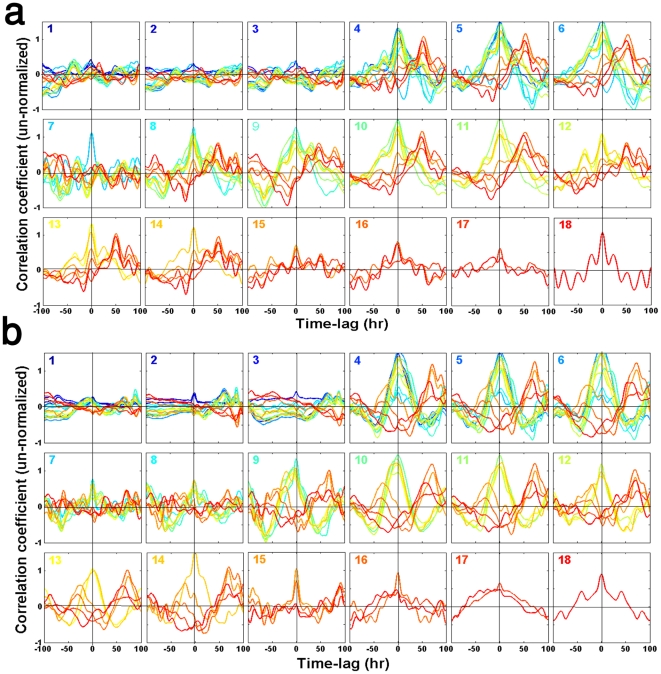
Cross correlation functions. The un-normalized cross correlation coefficient as a function of time-lags was computed between all the genes of populations (a) Ia and (b) Ib. The computed cubic-spline interpolation profiles for the high resolution data set of Fig. S2, was used to compute the cross correlations by direct summations (see *Methods*). The number in each plot is for a given gene (numbers the same order as in Fig. 1b; see *Methods*) which is cross-correlated with all other genes with higher numbering-label. The autocorrelation curve has the same color as the plot-number. The time-lags are measured in hrs, where 50 hrs correspond to ∼10 chemostat-dilution generations. As a control, randomly shuffled surrogate profiles showed flat correlation functions.

**Figure 7:**
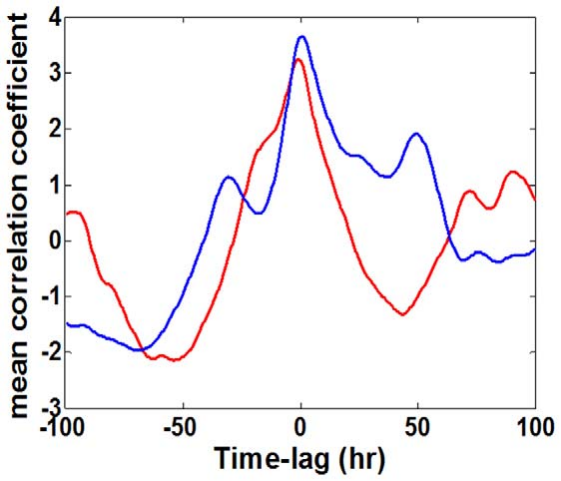
Mean cross-correlation coefficients. Based on the correlation functions shown in Figs. 6a,b, the figure presents the mean cross correlations as a function of time-lags. Each curve is the result of averaging the cross-correlation coefficients (including autocorrelations) over the entire set of gene pairs, for population Ia (blue) and population Ib (red).

We finally analyzed the dynamic modes in the expression response of the cell populations. Figs. 6a,b show the computed cross-correlations as a function of time-lags, between a given gene whose number labels the plot and all other genes labeled with higher numbers (numbering order is the same as in Fig. 1b and the detail list in the *Methods*) within the population, for populations Ia and Ib, respectively. The plots include the autocorrelations (shown in the same color as the plot-label). Fig. S5 shows similar long-term correlations for the inter-populations cross-correlations. The data show significant correlations at long time lags (>50 hrs ∼10 chemostat-dilution generations), quantifying the coherency of the expression dynamics over many cell generation times. Fig. 7 emphasizes the significance of these long-term correlations by showing the mean correlation coefficients as a function of time-lags, obtained by averaging the entire set of correlations in each time-lag, for the two populations of Figs. 6a,b. It clearly shows long-term relaxations of the zero-lag correlations as well as the emergence of significant peaks at time-lags 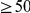 hrs. Such coherency over many cell generations suggests the involvement of underlying epigenetic processes.

### Expression response of “wild-type” cell populations

**Figure 8:**
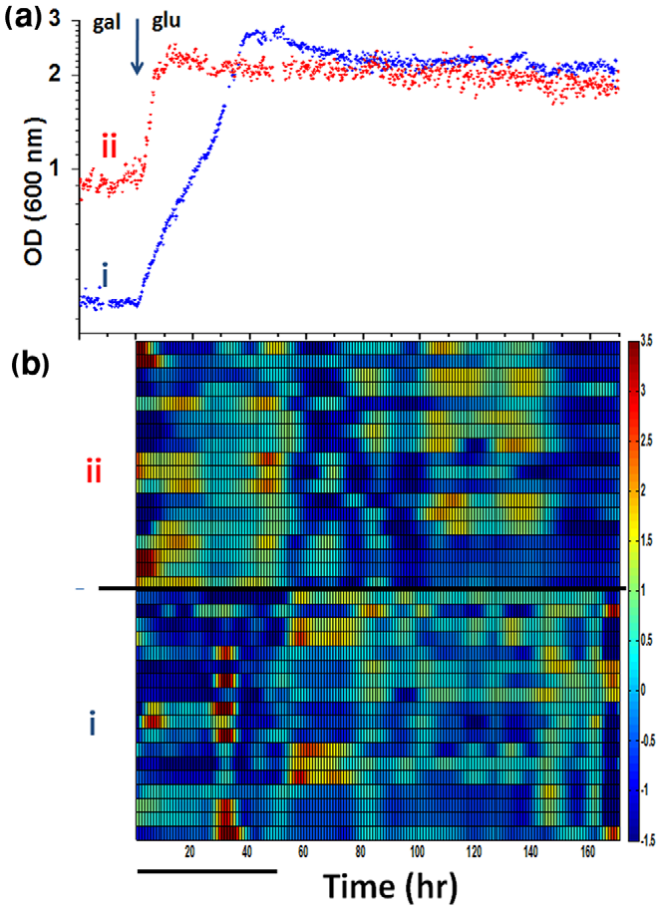
Phenotypes and gene expression profiles for “wild-type” cells. (a) Cell density (OD at 600 nm) as a function of time for two repeated chemostat experiments with populations of “wild-type” cells deleted of *HIS3*. The histidine-containing medium was switched from galactose to glucose as a sole carbon source at t = 0, leaving all other nutrients the same. A steady state was first established in galactose prior to this medium switch into glucose. Note the y-axis logarithmic scale. (b) Color-coded raster plot of the mRNA expression profiles for the two populations (i and ii) as in (a). The expression levels were measured for 18 genes belonging to different metabolic functional modules (see *Methods* for list of genes at the same order of appearance as in the figure, starting with *GFP* under pGAL10 as the first gene from the bottom). The measured expression levels were normalized for each gene to zero mean and unit standard deviation across its entire time profile. The color-coded profiles are cubic-spline interpolations of the measured data points shown in Fig. 9. Bar - 10 chemostat-dilution generations.

**Figure 9:**
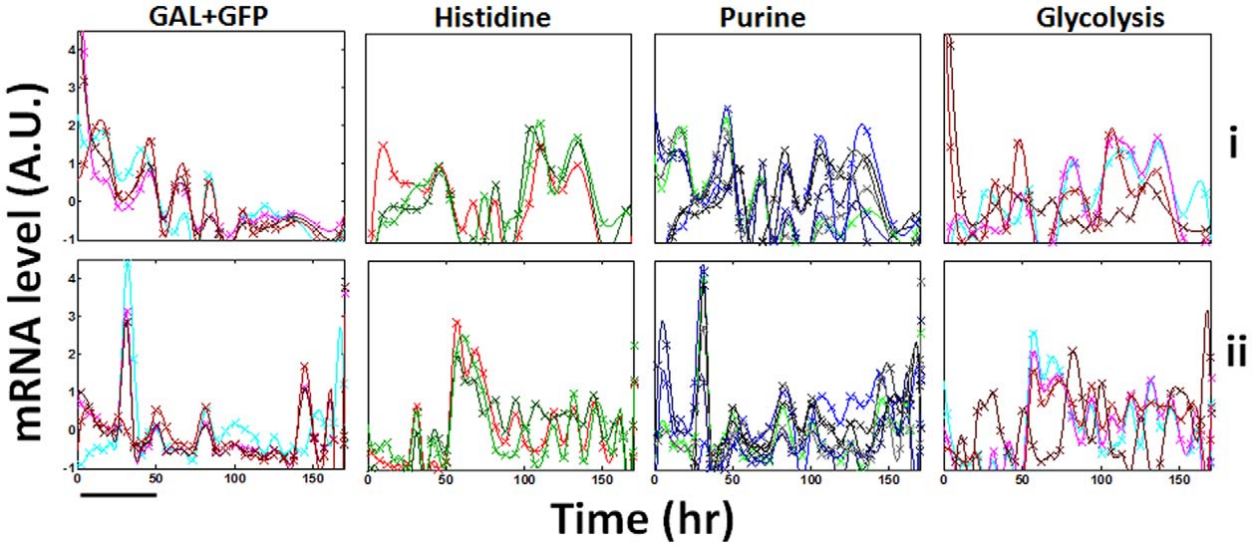
Normalized expression profiles for a “wild-type” strain. The normalized mRNA levels measured for cells deleted of the *HIS3* gene and grown in the same chemostat system as the rewired cells (medium supplied with histidine). The normalization is as in Fig. 8: The measured mRNA profile for each gene (relative to the value of *ACT1* at that time point) were normalized by subtracting the mean value and divided by the standard deviation; mean and standard deviation computed over the entire time period measured. (a) Population i and (b) population ii, as in Fig. 8. The medium was switched from galactose to glucose at t = 0. Bar: 10 chemostat-dilution generations.

For comparison, the population dynamics of “wild-type” cells deleted of the gene *HIS3*, grown in a chemostat at identical parameters as before and switched from galactose-based to glucose-based histidine-containing medium, were measured. Fig. 8a shows that the dynamics of two such populations (from two separate experiments), following the switch from galactose to glucose (t = 0), were significantly different from the ones emerging for the genome-rewired cells. Indeed, in contrast to the complex population growth dynamics of Fig. 1a, here the population cell density exhibited a fast exponential increase from one steady-state in galactose into a second, higher steady-state in glucose, since the latter is a more efficient carbon source. Note the significant differences between the dynamics of repeated populations which nevertheless converged to similar glucose steady states. We noted before that repeated chemostats with nominally identical parameters could stabilize at different galactose steady-state cell density levels (see Fig. 2 in [23]). Fig. 8b shows that similar to the rewired cells, “wild-type” cells also exhibited collective dynamics in the expression of the measured genes, with significant oscillatory peaks of activity some of which emerged long after the switch to glucose. The two repeated populations exhibited significantly different expression patterns. Fig. 9 shows the detailed profiles of expression for the different genes in the two populations divided according to their functional groups (compare to Fig. 2 for the rewired cells). Note again that the patterns of expression exhibited multiple modes and that not all the genes within a functional module exhibited identical patterns. Some of the genes in one population exhibited damped oscillatory modes of expression levels in direct response to the medium switch, while some other genes exhibited strong fluctuating dynamics. Damped oscillations might result from de-phasing of cells that initially responded in synchrony to the abrupt medium switch [29] but significant population-average activity peaks emerging in such large populations (∼10^9^ cells) not in direct response to the medium switch must result from synchronization of the expression response between the cells.

## Discussion

We have shown that each population of genome-rewired cells developed a unique pattern of gene expression, reflecting the collective population dynamics; an integrated outcome of intracellular and intercellular processes connected through transgeneration memory [30], [31], [32], [33]. The emerging patterns of expression of essential metabolic genes were significantly different between twin populations as well as for populations with non-jointed histories. A unique pattern of expression dynamics was also observed for each population of “wild-type” cells. Significant gene expression dynamics emerged also during periods of steady growth and apparent steady-state cell density. Importantly, the observed gene expression profiles for rewired as well as for “wild-type” cells exhibited multimode dynamics where each mode populated with a group of coherently responding genes from different functional modules. This behavior is markedly different from previously observed collective gene expression dynamics in cell populations which showed a global “rigid-body” response in which the entire genome oscillated due to metabolic oscillations [34], [35]. Higher statistics on populations would be required to assess the universal aspects of the expression dynamics in adapting populations and their actual relation to the adaptation process or to the metabolic state of the cell. It is clear from the results presented here that indeed the relationship between patterns of expression of essential genes and the actual metabolic (phenotypic) state of the population is complex. Deciphering the mapping between dynamic patterns of expression and the metabolic state requires the development of a technology allowing measurements of high statistics on chemostat populations, which is not yet available. Parallel measurements on a large number of twin populations having identical histories will open the road to a “statistical mechanics” approach at the population level of organization. We leave this fascinating issue for future research.

In our experiments, the metabolic state of adapted rewired cells, with the cell phenotype emerging at the end of phase II (see Fig. 1) enabling them to grow stably in glucose, was shown to be stable even through significant perturbations of the environment and even through repeated cycles of galactose-glucose media changes [21]. Thus, the growth phenotype for adapted cells in our chemostat populations converged to a stable homeostatic state. In light of the fact that the expression pattern was different in each adapting population, what processes did then determine such a stable phenotype of the cell? Similarly, “wild-type” populations converged to similar steady state densities in glucose and hence to similar metabolic phenotypes, in spite of the differences in their patterns of expression. The flexibility in expression response shows that tight regulation was not necessary for such stability and that different time-dependent patterns of gene expression could lead to similar phenotypic responses. In other words, the mapping from gene expression to the phenotype is highly degenerate. Indeed, the mapping between gene expression and a metabolic state is a dynamical process sensitive to initial conditions [18], [19], [36], where the former provides a non-specific envelope of response, an infrastructure support enabling convergence in the metabolic functional space. It emerges that gene expression should be considered more as an “auxiliary tool” for the cell rather than as a “programmed” determinant process. The freedom in the gene expression process however, did not reflect an intracellular stochastic process [37]; cell-to-cell variability within populations would be averaged-out in our measurements over such large populations (containing typically around 10^9^–10^10^ cells and at no time smaller than ∼10^7^–10^8^ cells). Thus, we conclude that the process of gene expression and the mapping between the vector of expressed genes and the cell phenotypic state are determined by population processes rather than resulting solely from intracellular mechanisms, such as the intrinsic response of genetic networks, their structural connectivity and their coupling to other intracellular processes. The population-average measurements shown in this work could not exclude a possible subpopulation structure. For example, it would be impossible to rule out at this stage, a general heterogeneous temporal response of cells in the population (e.g., phenotypic switching between states at variable times). Such variability in temporal response between cells would result in variable population-average dynamic patterns. However, since the emerging expression activity peaks were not in direct response to the environmental switch and since the coherent gene activity spanned many chemostat-dilution generations (>10), these patterns of activity must involve stable trans-generation propagation and collective population dynamics. These dynamics could be carried out by several large subpopulations, beyond single-cell stochasticity. The existence of such subpopulations however, does not affect our major conclusion and is left for future studies.

If the expression response indeed reflects population effects, what then made each population unique? An expression pattern that was coherent within a population but irreproducible between populations suggests that a dynamic environment could play a significant role in synchronizing and shaping the population-average expression response. Indeed, the immediate environment of the cells, in contrast to the nominal medium feeding the chemostat which is identical for all the populations, is unique for each population. We propose to think of the environment as a dynamical entity coupled to the population dynamics itself and serving as a common driving force of the cells, affecting the global population expression response. Intercellular coupling through direct signals such as diffusion of a small specific molecule can cause cell synchronization, but such a response is typically sensitive to cell density (e.g., “quorum-sensing” [38], [39]). This scenario is unlikely in our experiments, since the coherent response seems independent of cell density (which as shown in Fig. 1 could vary by two orders of magnitude without affecting the coherent expression activity profiles). A more realistic possibility in our case is cell coupling via common resources in the medium: although the external feeding medium is identical for all populations, each of the populations develops dynamically within the chemostat its own unique environmental niche [40], [41]. Even slight differences in extraction of ingredients from the medium or secretion of intracellular materials by the growing cells, can globally affect the population expression dynamics. This hypothesis can in principle be tested in future experiments, by mixing the extracellular media of two parallel adapting populations, each medium conditioned by its original population before the mixing, at critical stages of the dynamics and examining the changes in the temporal expression profiles.

Finally, the picture arising from our experiments suggests that gene expression is a self-organization process, in which the intracellular degrees of freedom are coupled through the environment to create a converging collective population dynamics. Although rather speculative at this stage, we believe that this behavior reflects a general organization principle. Cells are seldom growing in isolation and thus in most biologically relevant situations, the genotype-to-phenotype transition should be understood in a population context with the environment as a coupled dynamic variable [36], [41], [42]. It remains to be seen how general this behavior is and its applicability to other biological phenomena. A satisfactory theoretical framework for such a self-organizing system is still lacking and remains a challenge at the forefront of biophysics.

## Materials and Methods

### Strain and chemostat growth conditions

Experiments were carried out with the haploid yeast strain YPH499 [*Mat*a, *ura3-52*, *lys2-801*, *ade2-101*, *trp1-Δ63*, *his3Δ200*, *leu2Δ1*] carrying the plasmid vector pESC-LEU (Stratagene) containing the pGAL1-pGAL10 divergent promoter with *HIS3* under pGAL1 [21]. *his3Δ200* is a deletion that removed the entire *HIS3* coding region plus the upstream promoter region, including the *Gcn4* regulatory sequence. Cells were grown in homemade chemostats [21] in synthetic dropout medium lacking histidine and leucine with the appropriate amino-acid supplement and 2% of either pure galactose or pure glucose as a sole carbon source. Throughout the experiments, the sugar (either galactose or glucose) was always in excess (maximal consumption of the cells is 25% of the sugar fed). Medium (concentrations in g/l): 1.7 yeast nitrogen base without amino-acids and ammonium sulfate, 5 ammonium sulfate, 1.4 amino-acids dropout powder (without tryptophan, histidine, leucine and uracil; Sigma), 0.01 L-tryptophan, 0.005 uracil. Growth in the chemostat was limited by the concentration of the amino acid supplement. The control “wild-type” strain did not contain the *HIS3* gene on the plasmid and the medium was supplemented with histidine (0.005 g/l); all the other chemostat parameters were the same as for the rewired cells. Two identical chemostats were constructed and operated in parallel. Feeding was done from the same source. The two chemostats had a closed-loop line between them, allowing fast mixing of the cells via a separate pump. Steady state in galactose was established while mixing was done at a rate faster than the chemostat dilution rate. This mixing line was decoupled prior to the switch into glucose, but the feeding source stayed common throughout the experiment. Each chemostat had its own online measurement system [21] that was used to measure the optical density (OD) of cells in the chemostat. Each chemostat also had its own homemade cell collector [21] that was used to automatically collect samples of cells from the chemostat at precise time points along the experiment and instantaneously freeze them. These samples were used for the real-time PCR measurements. The chemostat generation time equals chemostat dilution time×ln2; ∼5 hr.

### mRNA measurements using real-time PCR

Total RNA was prepared from cells extracted from the chemostats at precise time points, by phenol extraction followed by cDNA preparation (oligo-d(T)_16_; TAQMAN-Reverse Transcription Kit, Applied Biosystems). Real-time PCR measurements were performed with AB 7700 (SYBR master mix, AB). A set of designed primers (Primer Express, AB;) was verified to work with uniform efficiency and led to the same quantitative results by using calibrated genomic DNA [21]. Measured amounts of *ACT1* prepared by PCR served as a ruler. In all measurements a non-template control for each of the primer pairs resulted in at least two orders of magnitude lower signal. All measurements were normalized by the *ACT1* transcription level measured in each sample as the other genes. Some of the measurements were performed in duplicates in the same PCR run and in most cases also repetitively in two separate PCR measurements. Typical measurement errors are shown in Fig. S3. In addition, measurement at time points close to each other serve as biological replicates since they were done on mRNA extracted from cells collected independently from the chemostat. Maximal errors were less than 3% in duplicates at the same PCR measurement and typically less than 15% between separate PCR measurements or separate RNA extraction samples from the same sample of cells. mRNA levels from 18 genes belonging to four different functional groups were measured as follows (gene order is identical to that numbered in all figures presented in the text): GAL system: *HIS3*, *GAL1*, *GAL2*; Histidine pathway: *His4*, *His7*, *His5*; Purine pathway: *YND1*, *IMD4*, *IMD3*, *ADE1*, *ADE12*, *ADE13*, *ADE17*, *ADE6*; Glycolysis: *CDC19*, *ENO2*, *GPM1*, *ADH1*.

The gene order for the “wild-type” cell measurements was similar with *HIS3* replaced with *GFP*, *GAL10* added and *ADE17* dropped out: GAL system: *GFP*, *GAL1*, *GAL2*, *GAL10*; Histidine pathway: *His4*, *His7*, *His5*; Purine pathway: *YND1*, *IMD4*, *IMD3*, *ADE1*, *ADE12*, *ADE13*, *ADE6*; Glycolysis: *CDC19*, *ENO2*, *GPM1*, *ADH1*. The *GFP* was located on the pESC-LEU plasmid under the pGAL10 promoter.

### Correlations and Bootstrap analysis

All computations were done using Matlab (MathWorks Inc.). The measured expression profiles over time were normalized for each gene to zero mean and unit standard deviation; the mean and standard deviation were computed for each gene from its entire temporal profile.

The Pearson correlation coefficient is defined as 
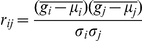
, where 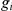 is the measured value of gene *i* and 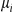 is the expectation value averaged over the entire set of measured time points, 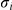 is the standard deviation of this entire set of time points for gene *i* and the bar denotes averaging over the entire set of time points. *j* denotes another gene measured from cells extracted from the same chemostat population as gene *i* or from a different population.

We used bootstrap resampling in order to approximate the distributions represented by the data and to compute statistics on each sample. For each gene pair in the data, the correlation coefficient was computed 1000 times by resampling the measured data, using random sampling with replacement while preserving the original number of data points. This bootstrap-produced data was then used to compute the average correlation coefficients and error-bars (standard deviations) for the correlations of a gene with itself in another population as shown in Fig. 4 and Fig. S4b.

The bootstrap resampled data was also used to compute the mean correlation coefficients between a gene *i* (equals the number on the x-axis) from a given population, and all other genes *j* within the same population and between populations, taking errors into account. The mean correlation coefficients shown in Fig. 5, were computed by: 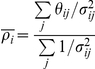 where 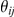 is the average correlation coefficient between gene *i* and gene *j* and 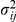 is the variance.

The cross correlation coefficients as a function of time-lags shown in Fig. 6 and Fig. S5 were computed as follows. We computed the cubic-spline interpolation, 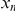, from the original data points measured along time for each gene, in order to smooth the data. The un-normalized correlation coefficient at time lag *m* was then computed by direct summation: 
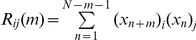
 for 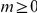, and 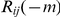 for 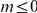, for genes *i* and *j* and *N* is the number of data points. We present un-normalized estimates for the correlations in Fig. 6 and Fig. S5 to illustrate the appearance of significant correlation peaks, compared to possible trivial correlations arising from gene profiles lacking significant dynamics. The normalized correlations show similar results.

## Supporting Information

Figure S1 **Un-normalized mRNA profiles.** The measured mRNA profiles for the same populations and functional groups as in Fig. 2 main text, not normalized by the mean and standard deviation.
(TIF)

Figure S2 **High resolution measurements.** The normalized mRNA profiles for the 18 genes for populations Ia and Ib from Fig. 1 main text measured at higher temporal resolution. The order of genes is (from top) GAL plus HIS3 (cyan), histidine group, purine group (divided arbitrarily to two subgroups for clarity) and glycolysis group. The order is the same as that specified in the *Methods*. The colors of the different gene profiles are the same as in Fig. 2 in the main text. Note that the main activity peaks are the same but higher frequency modes show up in the higher resolution data. These measurements also serve as biological “replicates” for some of the time points allowing us to assess the measurement errors.
(TIF)

Figure S3 **Real-time PCR measurement errors.** Some of the mRNA-level measurements for two of the populations, Ia (left panel) and Ib (right panel), were repeated to estimate the real-time PCR measurement errors. The upper graphs show the mean measured mRNA levels (normalized to *ACT1*) with their corresponding error-bars (standard deviations) while the lower graphs show the standard deviation over mean for the same data. The genes measured are: HIS3, h2-h5 histidine group, p5-p9 purine group and g3-g5 glycolysis group. The order of genes is the same as specified in the *Methods*.
(TIF)

Figure S4 **a: Correlation coefficient matrix for higher resolution measurements.** The Pearson correlation coefficient between the mRNA time profiles shown in Fig. S2, computed for all pair of genes for populations Ia and Ib of Fig. 1 main text. The correlation coefficients between genes within a population are near-diagonal pixels while inter-population ones are off-diagonal pixels. For each gene-pair the correlation coefficient is the result of averaging the correlations over the entire period shown in Fig. S2. **b:** Comparing the mean and error of correlation coefficients of a gene between populations for the high and lower resolution data. Mean and standard deviations of correlation coefficients computed between a given gene in one population and the same gene in another population. Bootstrap resampling (see *Methods*) was used to compute the mean and standard deviation (error bars) of the correlation coefficients for genes between the twin populations: Ia and Ib, for: (a) the same temporal resolution shown in Fig. 2 (red), and the higher resolution data of Fig. S2 (black). The gene number on the x-axis is at the same order as in Fig. 1b in the main text and corresponding to the list presented in *Methods*. The measured data points for each gene was resampled with replacement 1000 times.
(TIF)

Figure S5 **Cross correlation functions between populations.** The un-normalized cross correlation coefficient as a function of time-lags was computed between all the genes of population Ia and those of population Ib. The cubic-spline interpolation profiles for the high resolution data of Fig. S2, was used to compute the cross correlations by direct summations (see *Methods*). The number in each box is for a given gene (numbers the same order as in Fig. 1b in the main text; see *Methods*) which is cross-correlated with all other genes. The autocorrelation curve has the same color as the plot-number. The time-lags are measured in hrs, where 50 hrs correspond to ∼10 chemostat generations. As a control, randomly shuffled surrogate profiles showed flat correlation functions.
(TIF)
