# Supplementary figures and images for: Collective Dynamics of Gene Expression in Cell Populations
Source: PLoS One. 2011 Jun 15;6(6):e20530. doi: 10.1371/journal.pone.0020530 (PMC3115940; doi:10.1371/journal.pone.0020530)

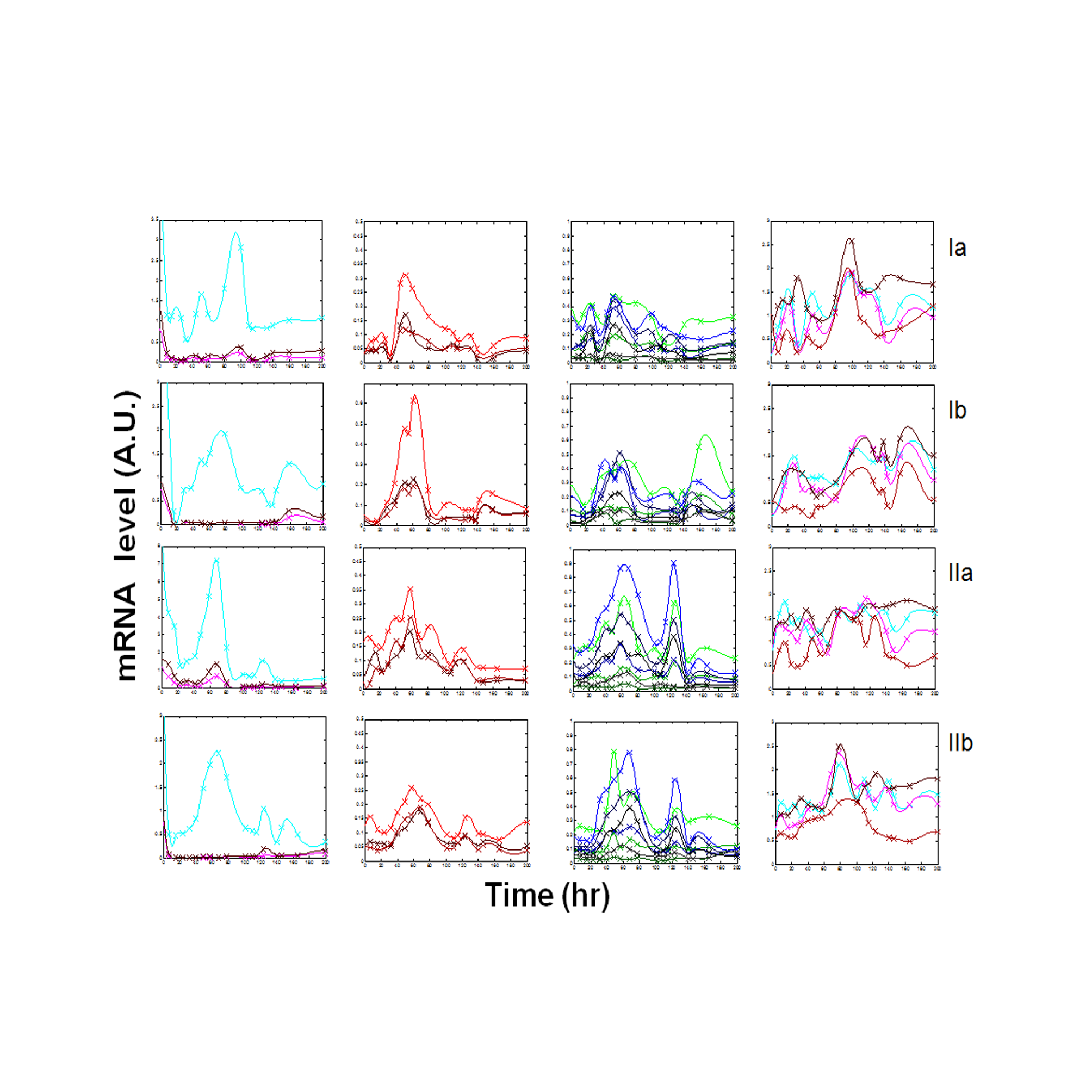

Supplement: Figure S1 — Un-normalized mRNA profiles. The measured mRNA profiles for the same populations and functional groups as in Fig. 2 main text, not normalized by the mean and standard deviation. (TIF) [file pone.0020530.s001.tif]

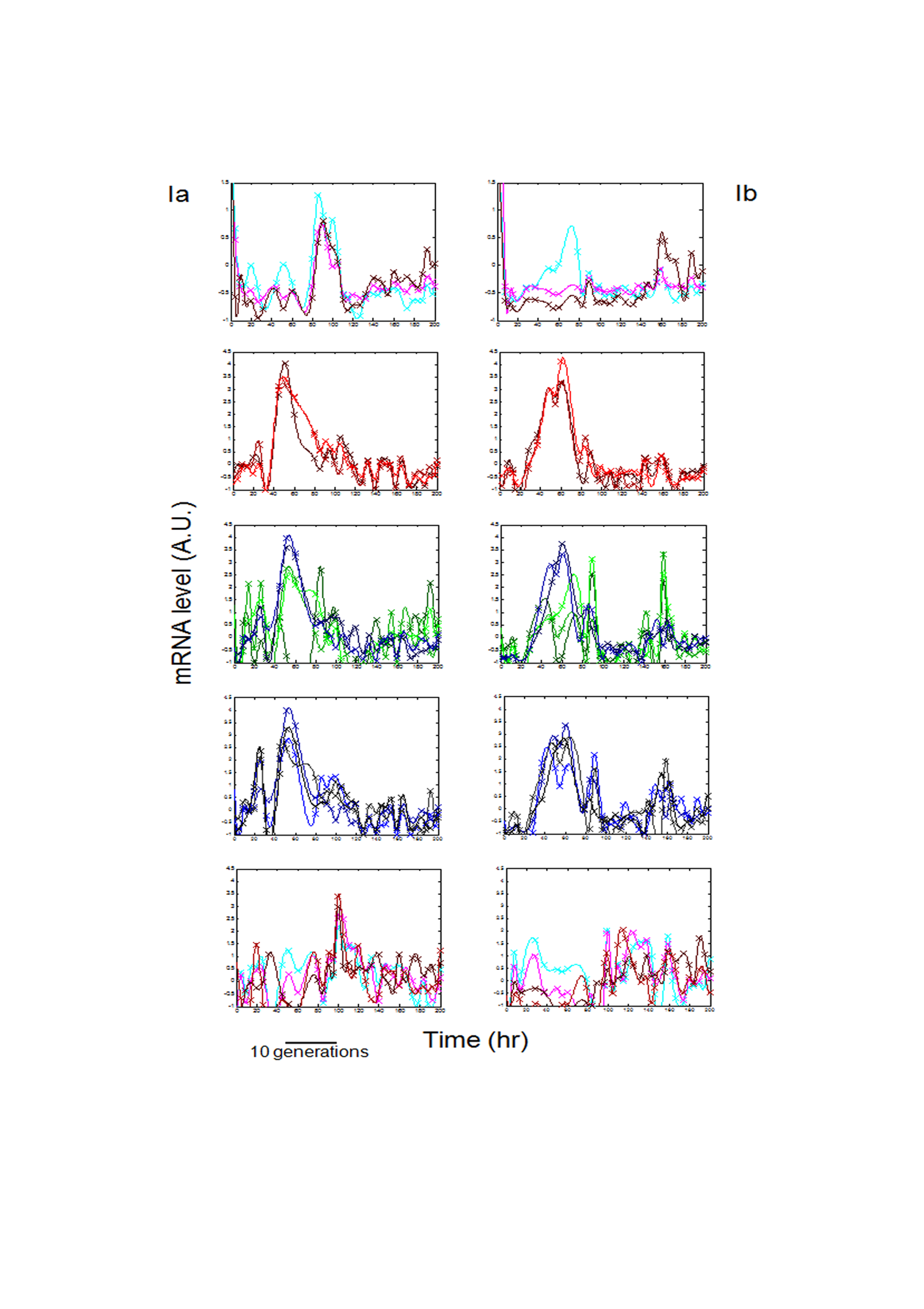

Supplement: Figure S2 — High resolution measurements. The normalized mRNA profiles for the 18 genes for populations Ia and Ib from Fig. 1 main text measured at higher temporal resolution. The order of genes is (from top) GAL plus HIS3 (cyan), histidine group, purine group (divided arbitrarily to two subgroups for clarity) and glycolysis group. The order is the same as that specified in the Methods. The colors of the different gene profiles are the same as in Fig. 2 in the main text. Note that the main activity peaks are the same but higher frequency modes show up in the higher resolution data. These measurements also serve as biological “replicates” for some of the time points allowing us to assess the measurement errors. (TIF) [file pone.0020530.s002.tif]

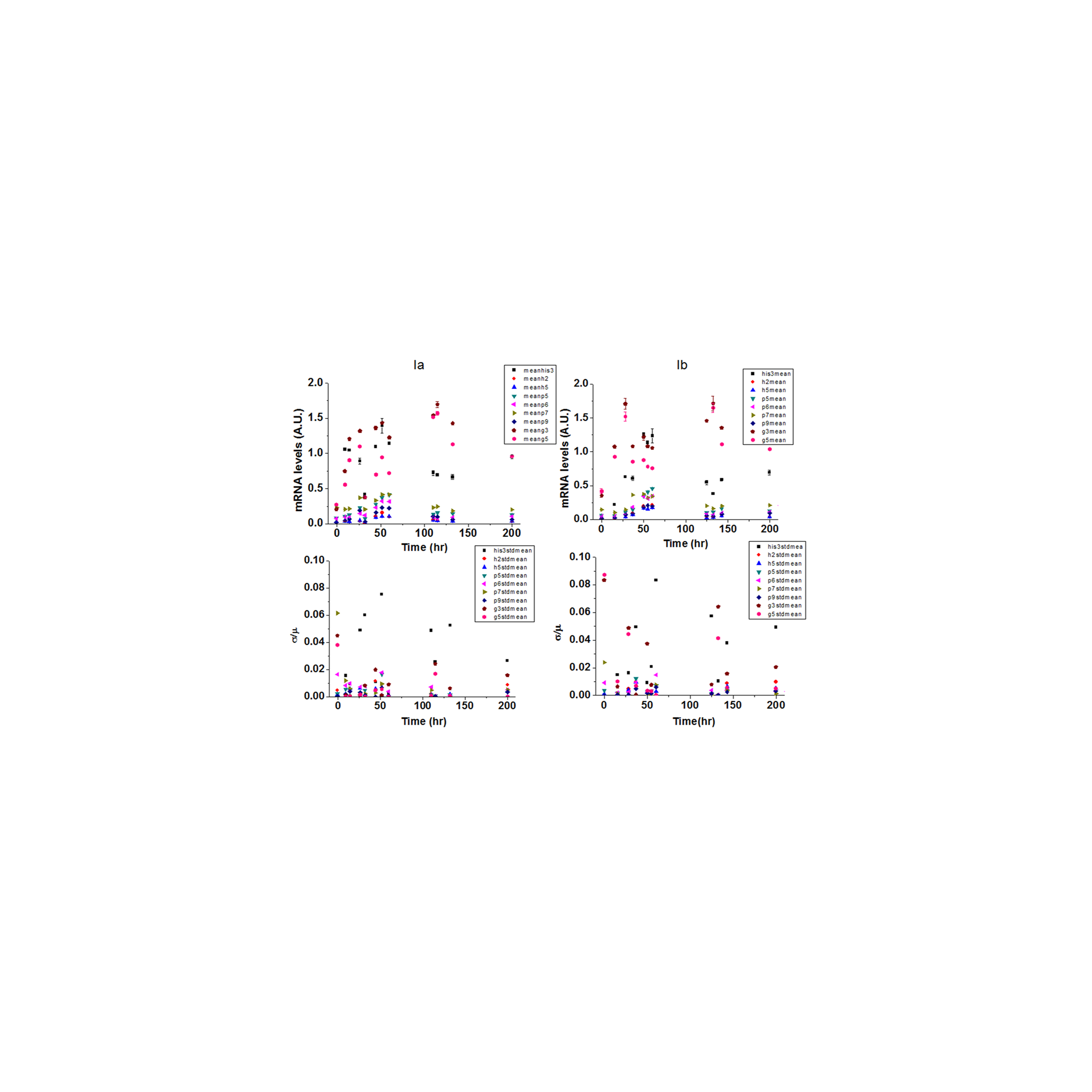

Supplement: Figure S3 — Real-time PCR measurement errors. Some of the mRNA-level measurements for two of the populations, Ia (left panel) and Ib (right panel), were repeated to estimate the real-time PCR measurement errors. The upper graphs show the mean measured mRNA levels (normalized to ACT1) with their corresponding error-bars (standard deviations) while the lower graphs show the standard deviation over mean for the same data. The genes measured are: HIS3, h2-h5 histidine group, p5-p9 purine group and g3-g5 glycolysis group. The order of genes is the same as specified in the Methods. (TIF) [file pone.0020530.s003.tif]

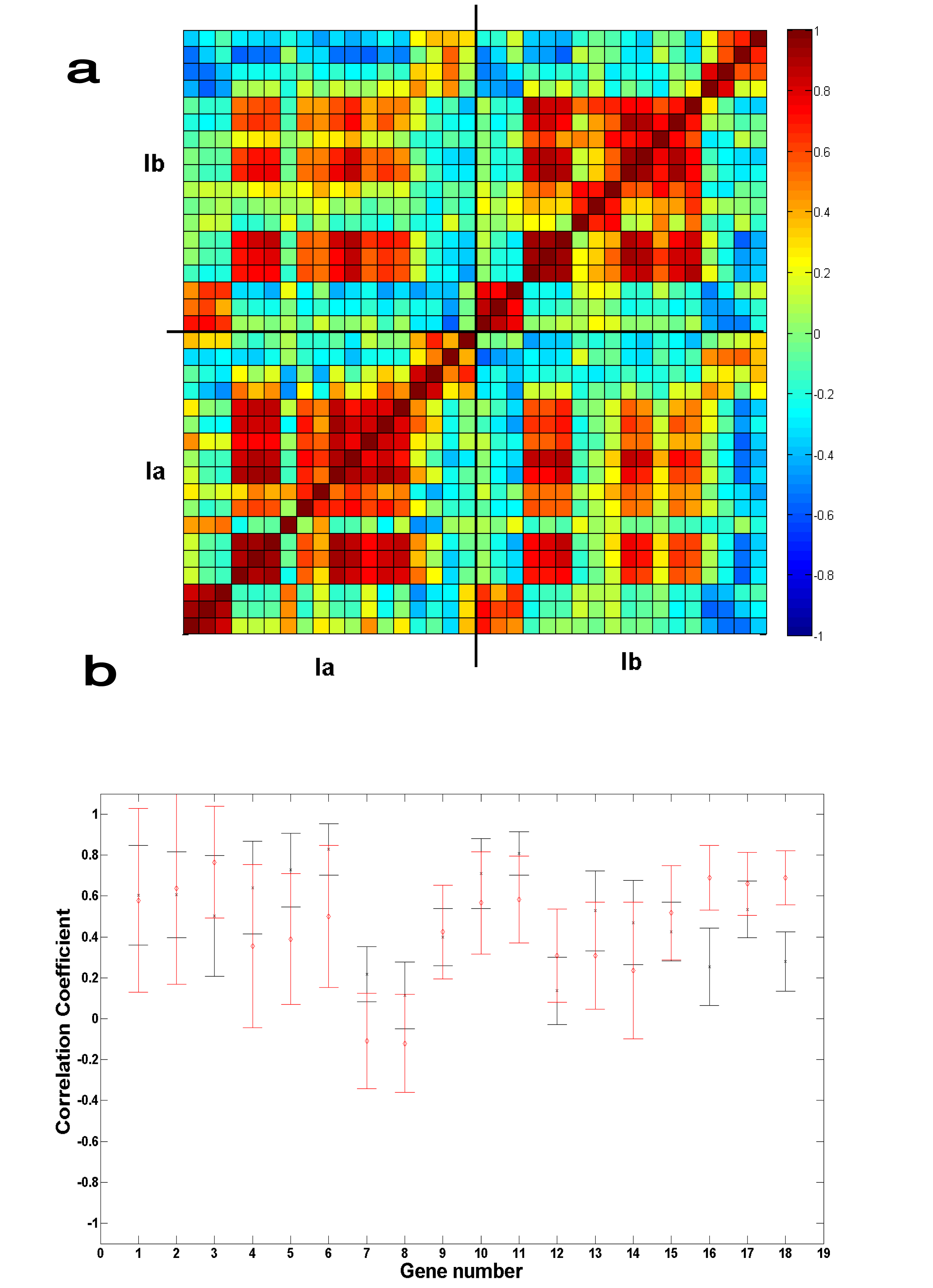

Supplement: Figure S4 — a: Correlation coefficient matrix for higher resolution measurements. The Pearson correlation coefficient between the mRNA time profiles shown in Fig. S2, computed for all pair of genes for populations Ia and Ib of Fig. 1 main text. The correlation coefficients between genes within a population are near-diagonal pixels while inter-population ones are off-diagonal pixels. For each gene-pair the correlation coefficient is the result of averaging the correlations over the entire period shown in Fig. S2. b: Comparing the mean and error of correlation coefficients of a gene between populations for the high and lower resolution data. Mean and standard deviations of correlation coefficients computed between a given gene in one population and the same gene in another population. Bootstrap resampling (see Methods) was used to compute the mean and standard deviation (error bars) of the correlation coefficients for genes between the twin populations: Ia and Ib, for: (a) the same temporal resolution shown in Fig. 2 (red), and the higher resolution data of Fig. S2 (black). The gene number on the x-axis is at the same order as in Fig. 1b in the main text and corresponding to the list presented in Methods. The measured data points for each gene was resampled with replacement 1000 times. (TIF) [file pone.0020530.s004.tif]

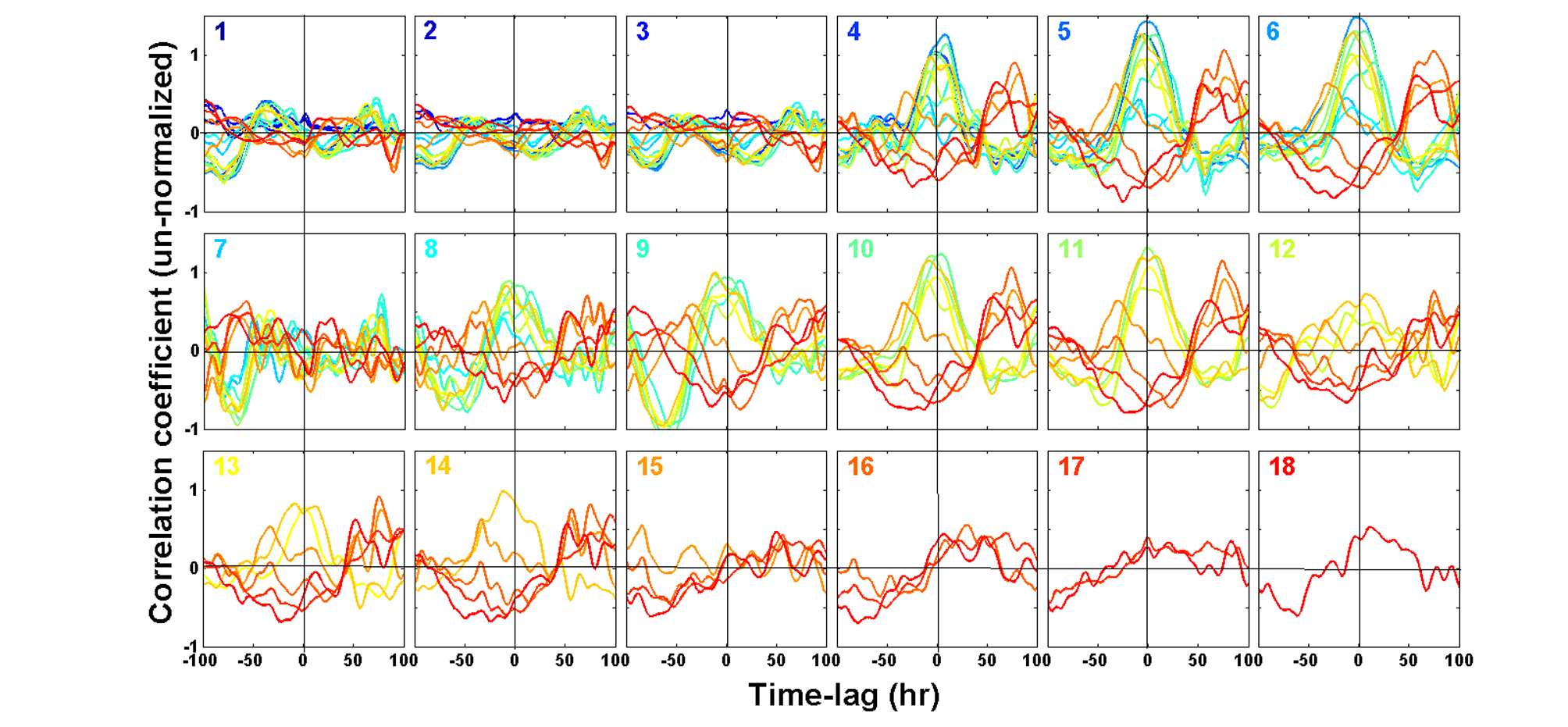

Supplement: Figure S5 — Cross correlation functions between populations. The un-normalized cross correlation coefficient as a function of time-lags was computed between all the genes of population Ia and those of population Ib. The cubic-spline interpolation profiles for the high resolution data of Fig. S2, was used to compute the cross correlations by direct summations (see Methods). The number in each box is for a given gene (numbers the same order as in Fig. 1b in the main text; see Methods) which is cross-correlated with all other genes. The autocorrelation curve has the same color as the plot-number. The time-lags are measured in hrs, where 50 hrs correspond to ∼10 chemostat generations. As a control, randomly shuffled surrogate profiles showed flat correlation functions. (TIF) [file pone.0020530.s005.tif]
